# Demographic trends in the incidence of young‐onset colorectal cancer: a population‐based study

**DOI:** 10.1002/bjs.11486

**Published:** 2020-03-09

**Authors:** A. C. Chambers, S. W. Dixon, P. White, A. C. Williams, M. G. Thomas, D. E. Messenger

**Affiliations:** ^1^ School of Cellular and Molecular Medicine University of Bristol Bristol UK; ^2^ Department of Colorectal Surgery University Hospitals Bristol, Bristol Royal Infirmary Bristol UK; ^3^ Department of Engineering Design and Mathematics University of West of England Bristol UK

## Abstract

**Background:**

Evidence is emerging that the incidence of colorectal cancer is increasing in young adults, but the descriptive epidemiology required to better understand these trends is currently lacking.

**Methods:**

A population‐based cohort study was carried out including all adults aged 20–49 years diagnosed with colorectal cancer in England between 1974 and 2015. Data were extracted from the National Cancer Registration and Analysis Service database using ICD‐9/10 codes for colorectal cancer. Temporal trends in age‐specific incidence rates according to sex, anatomical subsite, index of multiple deprivation quintile and geographical region were analysed using Joinpoint regression.

**Results:**

A total of 56 134 new diagnoses of colorectal cancer were analysed. The most sustained increase in incidence rate was in the group aged 20–29 years, which was mainly driven by a rise in distal tumours. The magnitude of incident rate increases was similar in both sexes and across Index of Multiple Deprivation quintiles, although the most pronounced increases in incidence occurred in the southern regions of England.

**Conclusion:**

Colorectal cancer should no longer be considered a disease of older people. Changes in incidence rates should be used to inform future screening policy, preventative strategies and research agendas, as well as increasing public understanding that younger people need to be aware of the symptoms of colorectal cancer.

## Graphical abstract

The incidence rate of colorectal cancer in young adults is increasing. Detailed epidemiological analysis of these trends is necessary to understand them better. This article shows that the rising incidence in young adults is being driven by distal colorectal tumours, with the magnitude of trends greatest in southern regions of England, independent of sex or socioeconomic status.

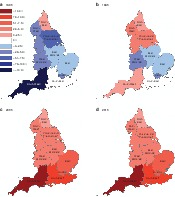

Incidence increasing in young adults

## Abstract (es)

**Antecedentes:**

Están apareciendo evidencias de que la incidencia del cáncer colorrectal (*colorectal cancer*, CRC) está aumentando en adultos jóvenes, pero se carece de la epidemiología descriptiva necesaria para comprender mejor estas tendencias.

**Métodos:**

Se realizó un estudio de cohortes de base poblacional de todos los adultos de 20 a 49 años diagnosticados de CRC en Inglaterra entre 1974 y 2015. Los datos se extrajeron de la base de datos NCRAS utilizando los códigos ICD9/10 para el CRC. Las tendencias temporales en las tasas de incidencia específicas por edad (*incidence rates*, IR) según el sexo, la localización anatómica, el quintil del índice de privación múltiple (*index of multiple deprivation*, IMD) y la región geográfica se analizaron mediante un modelo de regresión joinpoint.

**Resultados:**

Se analizaron un total de 56.134 nuevos diagnósticos de CRC. El aumento más sostenido en la IR se produjo en el grupo de edad de 20 a 29 años, principalmente a expensas de un incremento de los tumores distales. La magnitud de los aumentos de IR fue similar en ambos sexos y en los quintiles del IMD, aunque los aumentos más pronunciados en la incidencia se registraron en las regiones del sur de Inglaterra.

**Conclusión:**

El CRC ya no debe ser considerado una enfermedad de las personas mayores: los cambios en las tasas de incidencia deberán tenerse en cuenta en las futuras políticas de cribado, en las estrategias preventivas y en los proyectos de investigación, así como para aumentar la toma de conciencia de la población de que las personas más jóvenes deben estar al corriente de los síntomas del CRC.

## Introduction

Colorectal cancer is a major cause of cancer‐related mortality and is the third most common cause of cancer death in the UK^1^, ^2^. Advances in the surgical and oncological management of colorectal cancer are the most likely explanation for the UK age‐standardized mortality rate decreasing from 49 to 27 per 100 000 person‐years over the past 40 years^2^.

Despite age‐standardized incidence rates remaining static in the UK, as well as in other nations with a high human development index^3^, there is increasing evidence that incidence rates are increasing in adults aged less than 50 years. A US study^4^, using Surveillance, Epidemiology, and End Results data, revealed a doubling in the incidence rate of both colonic and rectal cancers among patients aged between 20 and 54 years since 1974. Similar findings have been demonstrated in cohorts from Canada^5^, ^6^, Australia^7^, New Zealand^8^ and, most recently, Europe^9^, suggesting that the underlying risk of colorectal cancer is increasing in young people.

Although men are well recognized to have a higher incidence of colonic and rectal cancer in older age groups, there is little difference in the incidence rates between men and women aged less than 40 years^10^, ^11^. UK data have shown that men have a higher proportion of rectal tumours, but that women have a higher proportion of right‐sided tumours^12^. However, data on anatomical subsite have not been linked to age‐specific incidence trends in the UK population. Data from North America^4^, ^6^ suggest that incidence rate increases have been driven by an increase in distal tumours, whereas European data^9^ suggest that incidence rate increases have been more pronounced for colonic cancer.

Socioeconomic status (SES) is associated with several important colorectal cancer risk factors^13^, ^14^, ^15^. In the UK, data from Northern Ireland^16^ have shown no difference in age‐standardized incidence between deprivation deciles, unlike in Scotland, where men from more deprived areas have an increased incidence of colorectal cancer with evidence of an increasing deprivation gap over time^17^, ^18^. Previous studies have focused on SES as a risk factor for colorectal cancer incidence, but this has never been analysed in the context of recent changes in age‐specific incidence trends in young adults. Significant variations in the burden of disease exist between the nine regions of England, including variation in the age‐standardized rate of years of life lost to colorectal cancer^19^, ^20^. Understanding whether there is a socioeconomic and regional variation in incidence rate trends in the young population could help elucidate potential aetiological factors.

Although data from the UK have been incorporated in recent Europe‐wide population‐based studies^9^, a more detailed description of the epidemiology underlying the recent increase in colorectal cancer incidence in young adults is required. This is vital, as young adults typically present with more advanced tumours that carry a poorer prognosis. A more thorough knowledge of the descriptive epidemiology would help inform future preventative strategies. Therefore, the aim of this study was to determine temporal trends in incidence of colorectal cancer stratified by sex, anatomical subsite in the colorectum, SES and geographical region of England.

## Methods

This study is reported according to the STROBE guidelines for epidemiological studies^21^. Data were obtained on all patients diagnosed with colorectal cancer aged 20 years and above from 1974 to 2015 using data from the National Cancer Registration and Analysis Service (NCRAS) (request ID: ODR1718_067). NCRAS is a UK‐wide partnership operated by Public Health England to collect data on all types of cancer, including colorectal cancer, occurring in the English population.

### Procedures

ICD codes were used to identify all diagnoses of colorectal cancer. ICD‐9 codes (colon 153.0–153.9 (excluding 153.5 – appendiceal tumour); rectum 154.0 and 154.1) for colorectal cancer were used for diagnoses made between 1974 and 1994. ICD‐10 codes (colon C18.0–C18.9 (excluding C18.1 – appendiceal tumour); rectum C19 (rectosigmoid) or C20 (rectum)) were used for diagnoses made between 1995 and 2015. Appendiceal adenocarcinomas were excluded and analysed separately (*Fig*. *S1*, supporting information). For the purposes of this study, young adults were defined as those aged 20–49 years, who were divided into three groups based on age at diagnosis: 20–29, 30–39 and 40–49 years.

Mid‐year population estimates were obtained from the Office for National Statistics (ONS) to provide population data stratified by age. Mid‐year population estimates in conjunction with the number of new diagnoses were used to calculate age‐specific incidence density rates per 100 000 person‐years, referred to hereafter as the age‐specific incidence rates, for each age group using the following.

$$\begin{array}{cc} & \text{Age‐specific incidence rate}\\ & \;=\frac{\text{No. of new cases in age group}}{\text{Mid‐year population estimate of age group}} \end{array}$$

The European Standard Population 2013 (ESP 2013) was then used to derive age‐standardized incidence rates for colonic and rectal cancer for the overall data set (20–49 years), in accordance with the methodology for direct standardization of the ONS^22^:

$$\begin{array}{cc} & \text{Age‐standardized incidence rate}\\ & \;=\frac{\sum (\text{ESP of age group}\times \text{age‐specific rate})}{\sum \text{ESP of age group}} \end{array}$$

Colorectal cancer cases were further stratified by sex (using sex‐specific population estimates from the ONS as above), anatomical subsite (proximal – caecum to descending colon; distal – sigmoid to rectum), geographical region (using region‐based population estimates from the ONS from 1981 onwards) and Index of Multiple Deprivation (IMD) quintile (from 2001 onwards). IMD is an area‐based metric that combines weighted information from seven domains: income (weighting 22·5 per cent), employment (22·5 per cent), education (13·5 per cent), health (13·5 per cent), crime (9·3 per cent), barriers to housing and services (9·3 per cent) and living environment (9·3 per cent). Lower‐layer super output areas (LSOAs; 32 844 in England) are given a value based on these domains. IMD quintiles were calculated by ranking all LSOAs from most to least deprived and then splitting this ranking into five equal groups; each quintile has 20 per cent of the ranked areas.

## Statistical analysis

Joinpoint Regression Program version 4.7.0.0 (National Cancer Institute; https://surveillance.cancer.gov/joinpoint/)^23^ was used to analyse the magnitude and direction of temporal trends in age‐specific incidence rates according to sex, anatomical site, IMD quintile and geographical region. Permutation analysis of the log‐transformed incidence rates was used to fit a series of joined lines with a minimum of 0 and a maximum of 5 join points. A series of comparisons among fitted models ranging from 0 to 5 join points was then undertaken to select the best‐fit model. This procedure allowed estimation of the annual percentage change (APC) in incidence. The squared correlation coefficient (*R* ^2^) was used to estimate the goodness of fit of the Joinpoint regression models to provide an indication of the extent of agreement between modelled and observed values. Inspection of residuals under the models presented here did not give cause for concern; standard errors appeared homoscedastic, free from serial correlation and without any unduly influential observations.

Age–period–cohort modelling (National Cancer Institute; Age Period Cohort web tool; https://analysistools.nci.nih.gov/apc) was used to assess the independent effects of age, time period and cohort on colorectal cancer incidence rates^24^. This was performed for all adults aged over 20 years. Three 10‐year age groups (20–29, 30–39 and 40–49 years for Joinpoint regression modelling) and four 10‐year periods (1976–1985, 1986–1995, 1996–2005, 2006–2015) were used for age–period–cohort modelling as it was necessary to have age and time‐period groups covering an equal timespan. Therefore, there were 11 birth cohorts starting in 1886 through to 1986 in 10‐year bands. Reference values for the age–period–cohort model were chosen arbitrarily from the first cohort analysed (1976–1985). Data presented from this model were shown as incidence rate ratios (IRRs) with 95 per cent confidence intervals to assess cohort effects. Local drift was estimated by examining age‐specific net APC in incidence rates.

## Results

Of the 1 145 639 new cases of colorectal cancer diagnosed between 1974 and 2015 in adults aged over 20 years, there were 2594 in 20–29 year olds, 11 406 among 30–39 year olds and 42 134 in 40–49 year olds.

### Age‐specific trends according to sex

**Figure 1:**
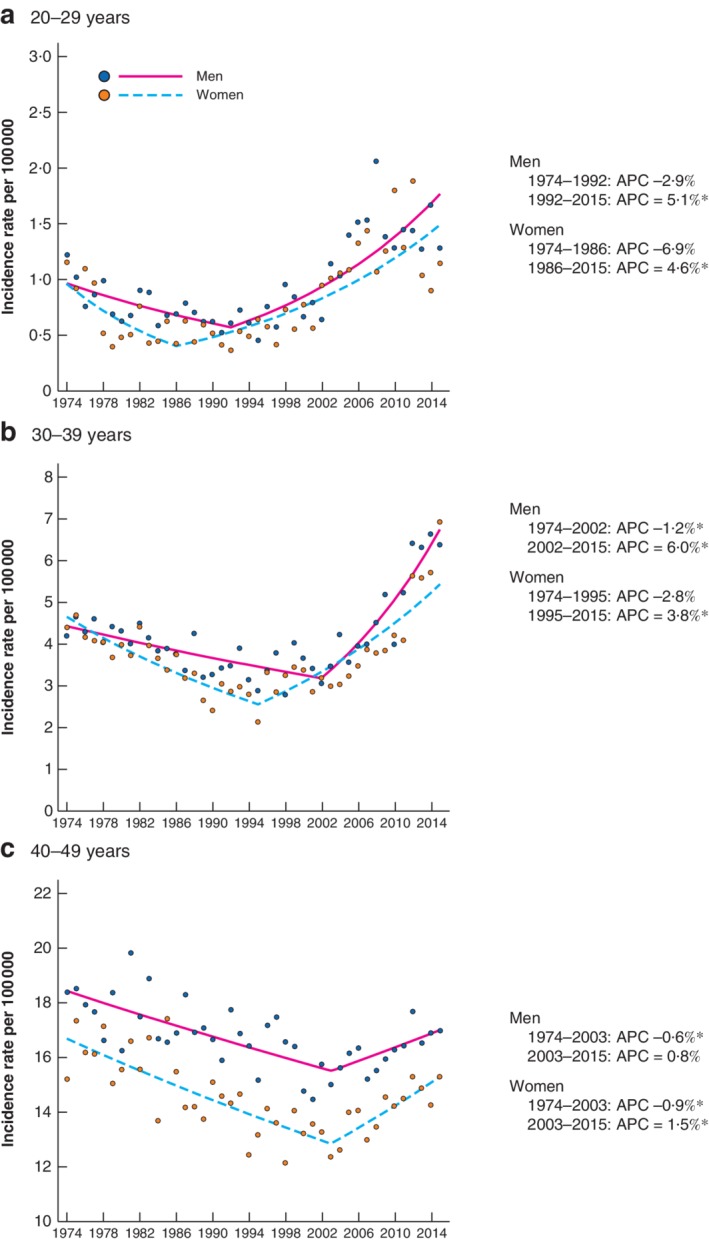
Age‐specific incidence of colorectal cancer in England stratified by sex: 1974–2015 Age‐specific incidence rates for men and women: **a** 20–29 years, **b** 30–39 years and **c** 40–49 years. Incidence rates per 100 000 are shown for each year; the range of values plotted on the *y*‐axis varies according to magnitude of incidence rate, to highlight trends. Plotted lines indicate annual percentage changes (APCs). *Significant change in APC *versus* 0 (*P* < 0·050) using the permutation model of logarithmically transformed data.

After an initial reduction in colorectal cancer incidence rates, there was a marked increase in rates among both 20–29 and 30–39 year olds. In 20–29 year olds, incidence rate increases commenced earlier in women (APC 4·6 (95 per cent c.i. 3·3 to 5·9) per cent from 1986) than in men (APC 5·1 (3·7 to 6·5) per cent from 1992) (*Fig*. 1 *a*). In 30–39 year olds, incidence rate increases commenced a decade later than in 20–29 year olds, with increases again being observed earlier in women (APC 3·8 (2·9 to 4·8) per cent from 1995) than in men (APC 6·0 (4·4 to 7·6) per cent from 2002). The incidence rate trends observed in the younger age groups were more attenuated in 40–49 year olds, with small increases observed from 2003 onwards in both women (APC 1·5 (0·5 to 2·5) per cent) and men (APC 0·8 (–0·1 to 1·6) per cent) (*Fig*. 1 *c*). These findings were suggestive of an age cohort effect and assessed in more detail using age–period–cohort modelling applied to the entire adult population aged over 20 years. Using the 1926 birth cohort as the reference group, the IRR of colorectal cancer for cohorts born from 1886 to 1966 remained constant, following which there was a progressive increase for successive birth cohorts (1976 cohort: IRR 1·4, 95 per cent c.i. 1·1 to 1·8; 1986 cohort: IRR 2·2, 1·3 to 3·8) (*Fig*. *S1* *b–k*, supporting information).

### Age‐specific trends according to anatomical subsite

**Figure 2:**
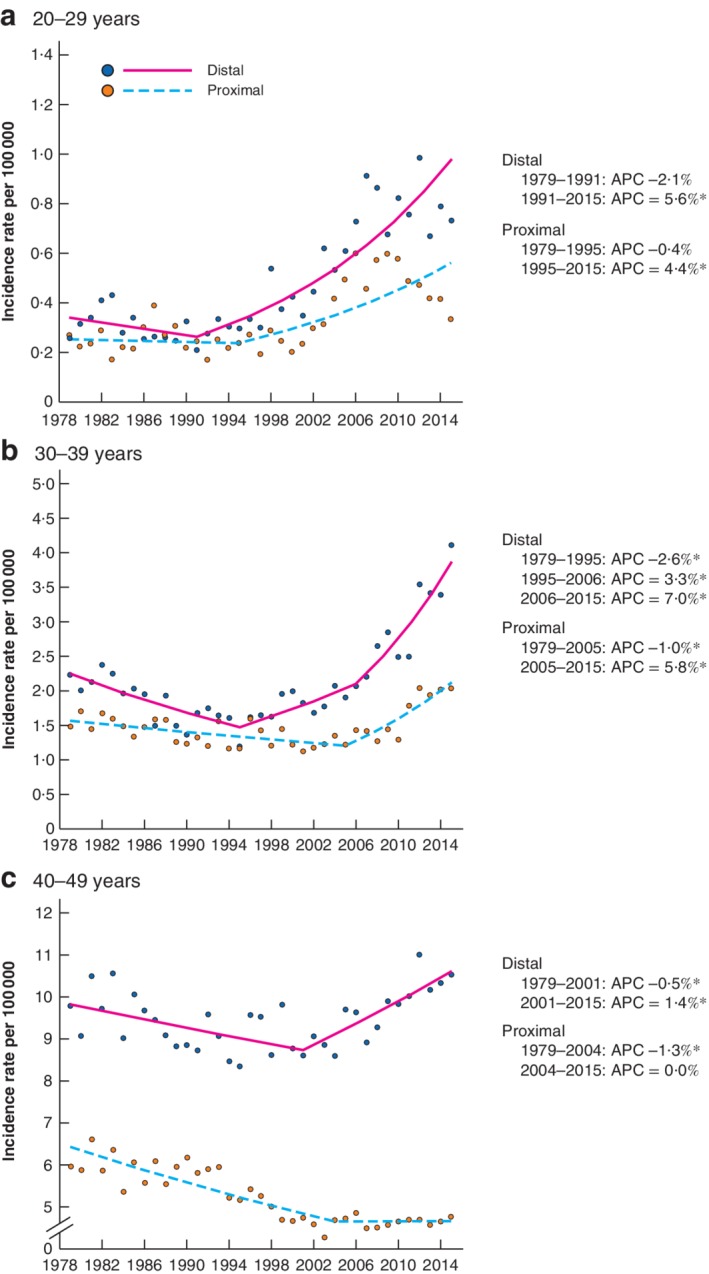
Age‐specific incidence of colorectal cancer in England stratified by tumour site: 1979–2015 Age‐specific incidence rates for proximal and distal tumours: **a** 20–29 years, **b** 30–39 years and **c** 40–49 years. Incidence rates per 100 000 are shown for each year; the range of values plotted on the *y*‐axis varies according to magnitude of incidence rate, to highlight trends. Plotted lines indicate annual percentage changes (APCs). *Significant change in APC *versus* 0 (*P* < 0·050) using the permutation model of logarithmically transformed data. Data from 1974 to 1978 were excluded as it was not possible to map ICD‐8 codes completely to later versions.

Increases in proximal cancer incidence rates were noted in 20–29 year olds (APC 4·4 (95 per cent c.i. 2·3 to 6·5) per cent from 1995) and 30–39 year olds (APC 5·8 (3·3 to 8·3) per cent from 2005), but with no observed effect in 40–49 year olds (APC 0·0 (–1·1 to 1·1) per cent from 2004) (*Fig*. 2). The increase in age‐standardized incidence rates of proximal cancer among 20–49 year olds was predominantly driven by increases in the incidence of caecal and ascending colon cancers (*Fig*. *S2*, supporting information).

Age‐specific incidence rate increases in distal cancers were more sustained and of greater magnitude than those for proximal cancers among 20–29 year olds (APC 5·6 (4·4 to 6·8) per cent from 1991) and 30–39 year olds (APC 3·3 (1·0 to 5·7) per cent from 1995 to 2006; APC 7·0 (4·2 to 9·8) per cent from 2006). A less pronounced increase in distal cancer was also noted among 40–49 year olds (APC 1·4 (0·7 to 2·1) per cent from 2001).

### Age‐standardized trends according to socioeconomic status

The age‐standardized incidence rates for distal cancers increased more rapidly than those for proximal cancers in all IMD quintiles, except quintile 2 (*Fig*. *S3* *a–e*, supporting information). There was no significant difference in the magnitude of incidence rate increases across the quintiles for either proximal (*P* = 0·110) or distal (*P* = 0·230) cancers.

### Age‐standardized trends according to geographical region

**Figure 3:**
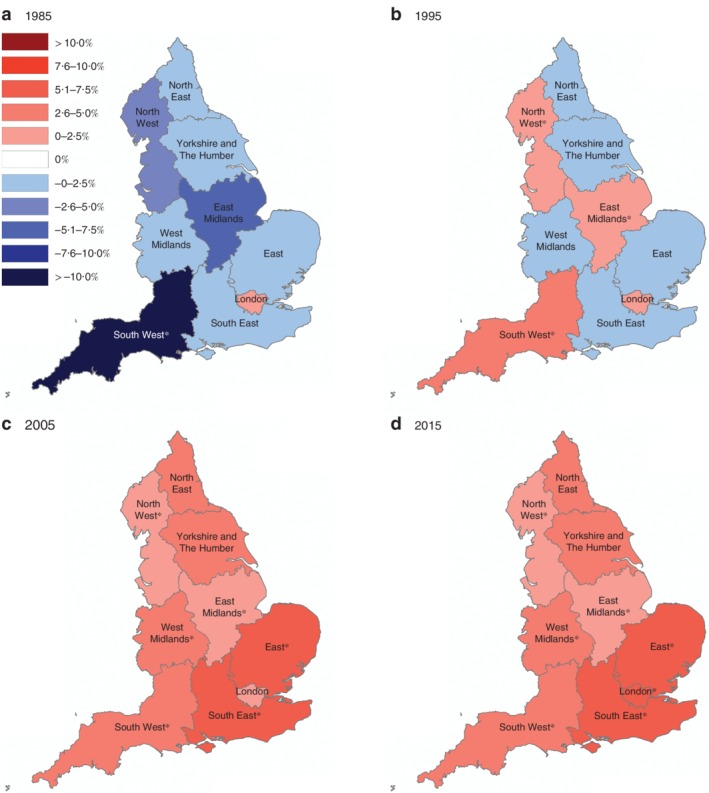
Regional variation in proximal colorectal cancer incidence rates: 1985–2015 Annual percentage change (APC) in proximal colorectal cancer incidence rates calculated for each region of England using Joinpoint regression analysis: **a** 1985, **b** 1995, **c** 2005 and **d** 2015. *Significant change in APC *versus* 0 (*P* < 0·050) using the permutation model of logarithmically transformed data.

**Figure 4:**
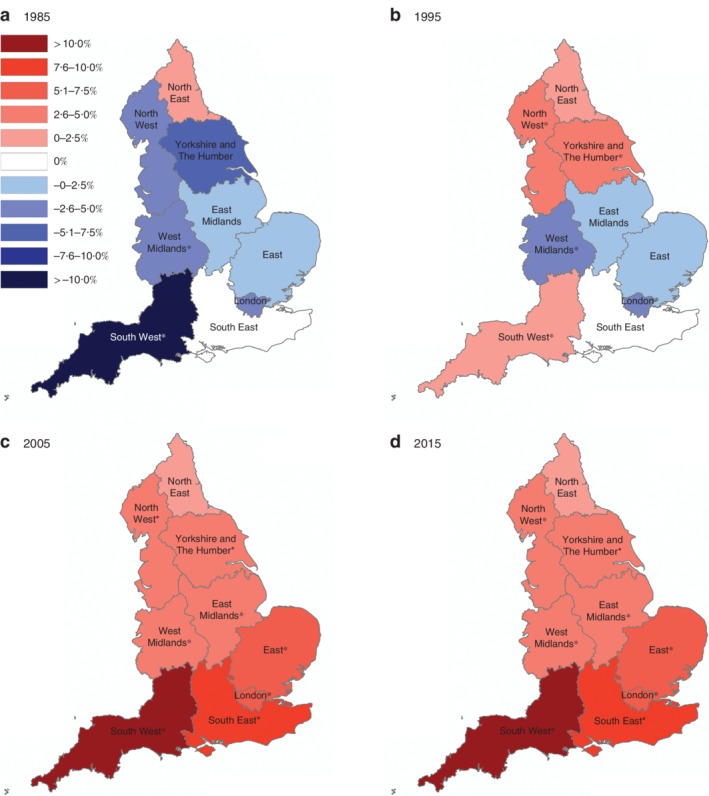
Regional variation in distal colorectal cancer incidence rates: 1985–2015 Annual percentage change (APC) in distal colorectal cancer incidence rates calculated for each region of England using Joinpoint regression analysis: **a** 1985, **b** 1995, **c** 2005 and **d** 2015. *Significant change in APC *versus* 0 (*P* < 0·050) using the permutation model of logarithmically transformed data.

In 1985, age‐standardized incidence rates of proximal cancers among 20–49 year olds were decreasing across all regions of England, except in London, with the greatest reduction observed in the South West (APC –12·1 (95 per cent c.i. –20·3 to –3·1) per cent (*Fig*. 3). By 2015, incidence rates were increasing fastest in the south‐eastern regions (South East: APC 7·4 (4·8 to 10·1) per cent; London: APC 6·5 (0·1 to 13·2) per cent; East: APC 6·0 (2·5 to 9·7) per cent). A similar, but more pronounced, trend was noted for distal cancers (*Fig*. 4). By 2005, the most rapid increase in age‐standardized incidence rates of distal cancer was noted in the South West (APC 10·1 (6·1 to 14·1) per cent); all other southern regions experienced annual increases of more than 5 per cent.

## Discussion

This large study based on a single, national population registry describes detailed epidemiological changes in colorectal cancer incidence in a young adult population. The finding that the incidence of colorectal cancer is increasing rapidly in young adults supports recent findings from other nations with a high human development index^4^, ^5^, ^6^, ^7^, ^8^, ^9^, ^25^. Rapid increases were observed in adults aged 20–39 years, which appear to be driven by increases in the rate of distal tumours. Incidence rate increases in the English population appear to be similar in both sexes and across all socioeconomic groups. Importantly, incidence rates have increased the fastest in the southern regions of England, particularly in the South West, where the incidence of distal cancers is now increasing by more than 10 per cent each year. A substantial birth cohort effect was observed, with dramatic increases in IRRs from the mid‐1960s onwards. This is similar to the observations in North American studies^4^, ^6^, although IRR increases in these studies appeared to have occurred in birth cohorts born approximately 15 years earlier. This suggests that any exposure to underlying risk factors may have occurred earlier in the North American population.

Tumours in young adults are thought to be sporadic^26^, with environmental factors likely to play a significant causative role. The rising incidence of colorectal cancer in young adults coincides with several environmental changes, most notably increasing childhood and adult obesity rates^27^. It is recognized that obesity in early life leads to an increased risk of developing colorectal cancer^28^, ^29^. Therefore, the increases in colorectal cancer incidence in young men and women may reflect the recent obesity trends in the UK, where prevalence rates among adults aged 35–54 years have increased from 15·4 to 26·3 per cent in men, and from 17·9 to 24·5 per cent in women, between 1993 and 2004^30^.

The more pronounced increase in the incidence rate of distal compared with proximal tumours contrasts with findings from recent European data^9^, but is similar to the results from several North American studies^4^, ^6^, ^31^. Although risk factors associated with an increased risk of colorectal cancer have been identified, the strength of their association with tumour development at individual sites in the colorectum remains unclear. Differences in the way environmental factors promote tumorigenesis at various sites in the colorectum suggest that proximal and distal tumours may be biologically distinct entities^32^; this may explain why the incidence in distal tumours among this English cohort has increased more rapidly. The biological differences in early‐ *versus* late‐onset colorectal cancer have been explored in several studies. A recent large cohort study^33^, ^34^ characterizing the clinical and molecular features of early‐onset colorectal cancer demonstrated enrichment of certain phenotypes, such as consensus molecular subtype 1 (CMS1) in distal tumours among adults aged less than 50 years. Other work^35^, ^36^ has shown low levels of microsatellite instability in colorectal cancer in young adults. Additionally, there is a prevalence of mutations in genes such as β‐catenin^35^, ^37^ and *KRAS* 38. Interestingly, the combination of altered environmental exposures and different tumour biology suggests that young‐adult colorectal cancer may be a different disease from later‐onset disease.

This study provided no evidence of an association between SES and the rate of increase in incidence of either proximal or distal tumours, in contrast to previous studies^16^, ^17^, ^18^ where higher incidence rates were observed in more deprived groups. Although factors associated with an increased risk of colorectal cancer, such as obesity, low‐fibre diet and reduced physical activity, are known to be associated with lower SES^1^, ^2^, ^3^, ^4^, ^5^, ^6^, ^7^, ^8^, ^9^, ^10^, ^11^, ^12^, ^13^, ^14^, ^15^, changes in obesity prevalence trends are actually similar between socioeconomic groups^39^; this may partly explain the lack of association between SES and colorectal cancer incidence rate increases in the present study. Furthermore, obesity is one of many risk factors associated with the development of colorectal cancer, and is itself caused by several complex societal, genetic and environmental interactions. It is perhaps not surprising that understanding the causative effects of single environmental risk factors is challenging^40^.

Geographical inequalities in health have been well characterized in England. Incidence rates of all cancers are higher in the north of England than in the south, although there is minimal variation in colorectal cancer incidence by region^41^. Recent incidence rate increases in colorectal cancer were observed across all English regions in the present study, although the most marked increases occurred in the south. It is difficult to explain why incidence rates are increasing more rapidly in young adults in the south given that risk factors such as obesity are increasing faster in northern regions^39^. It is important to point out that the effect of regional variations in access to healthcare/endoscopy services on colorectal cancer incidence rates remains unknown, and it may be that the incidence rate increases seen in the more affluent, southern regions have been driven by increased awareness and access to medical care.

The main strengths of this study are the size and completeness of the data set. Data were obtained from NCRAS, a nationally curated cancer registry, with 100 per cent complete data for 1974–2012 and 98·4 per cent complete data for 2013–2015. Unfortunately, stage‐specific data were not recorded routinely until 2012, so further analysis of incidence rate trends according to tumour stage was not possible. It is important to know whether the increase in young‐onset colorectal cancer has been driven by an increase in the detection of early‐stage disease, particularly in regions and socioeconomic groups that may have increased health awareness and access to endoscopy services. Data presented in this study are population‐based and specific causal inferences cannot be made. In addition, IMD quintile and geographical region are group‐level metrics and are unable to account for individual‐level contextual effects that could have affected the association between these variables and colorectal cancer incidence rates. Finally, endoscopy is being used increasingly in England^42^, and it could be argued that this accounted for the rising incidence of colorectal cancer. However, detection bias is unlikely as incidence rates were decreasing until the 1990s, and the most rapid increases were noted in the youngest age groups which are the least likely to attend for endoscopic examination.

The incidence rate of young‐onset colorectal cancer is increasing, particularly among adults aged 20–39 years. This trend appears to be predominantly driven by a rise in distal tumours. Incidence rate increases of a similar magnitude have been observed in both sexes and across IMD quintiles, but are most pronounced in the south of England. Importantly, there is a strong birth cohort effect and it is likely that the increased risk in the youngest cohorts will be carried forward as they age, which will place a significant burden on future healthcare resources. The role of environmental factors, such as diet, obesity, physical exercise and the gut microbiota, in the development of young‐onset colorectal cancer is incompletely understood and requires further research. Reducing the screening age to below 50 years will have significant resource implications in the current economic climate^43^ and, instead, there should be more focus on risk‐stratifying symptomatic younger patients to further investigation using tests such as quantitative faecal immunohistochemical testing.

## Supporting information


**Fig. S1 Colorectal cancer and appendiceal cancer incidence trends in England: 1974 to 2015** A Age‐standardised rates of appendiceal adenocarcinoma in England 1979‐2015. Appendix adenocarcinomas were identified using ICD9 code C153·5 (1979‐1994) and the ICD10 code C181 (1995‐2015) with no data available prior to 1978. Data shows increasing age‐standardised incidence of appendiceal cancers in all agegroups. B‐K Age‐period‐cohort analysis of colorectal cancer excluding appendiceal cancers. B Statistically significant birth cohort effects were observed with cohorts born after 1986 at 2·2 fold (95% CI 1·3‐3·8; X2 = 70·8, p < 0·001) increased risk of CRC compared to earlier cohorts. C Local drift of incidence rates are observed to be significantly different from net drift using Wald Test for heterogeneity (X2 = 36·3, p < 0·001) suggesting a single summary age‐standardised rate of CRC is inadequate to describe temporal trends in all age‐groups. D‐K Additional descriptive metrics from the age‐period‐cohort tool (National Cancer Institute's Age Period Cohort web tool, https://analysistools.nci.nih.gov/apc).
**Fig. S2 Age‐standardised incidence of colorectal cancer stratified by anatomical subsite: 1979 to 2015** The age‐standardised incidence of all anatomical subsites of colorectal cancer from 1979‐2015. Anatomical subsites were mapped between different ICD versions (ICD9 mapped to ICD10) for all colon and rectal codes (C18, C19 and C20). Data available from 1979 onwards as ICD8 data (1974‐1978) is not subdivided by specific anatomical site. Age‐standardised incidence is given as rate per 100 000 population.
**Fig S3. Age‐standardised incidence of colorectal cancer in adults aged 20‐49 years stratified by Index of Multiple Deprivation (IMD) quintile: 2001‐2015.** Annual percentage change (APC) of age standardised incidence for each IMD quintile (A ‐ Least deprived to E ‐ Most deprived) and for anatomical location (proximal or distal), was calculated with an asterisk showing statistically significant differences in APC from zero with p<0.05 using the permutation model of logarithmically transformed data. Regression lines were analysed using Analysis of co‐variance (ANCOVA) and revealed no evidence of a difference in incidence rate increases between IMD quintiles for either proximal (p=0.110) or distal (p=0.230) cancers.
