# Supplementary material for: Demographic trends in the incidence of young‐onset colorectal cancer: a population‐based study
Source: Br J Surg. 2020 Mar 9;107(5):595–605. doi: 10.1002/bjs.11486 (PMC7155067; doi:10.1002/bjs.11486)
Supplement: Supplementary file 1 — Fig. S1 Colorectal cancer and appendiceal cancer incidence trends in England: 1974 to 2015 A Age‐standardised rates of appendiceal adenocarcinoma in England 1979‐2015. Appendix adenocarcinomas were identified using ICD9 code C153·5 (1979‐1994) and the ICD10 code C181 (1995‐2015) with no data available prior to 1978. Data shows increasing age‐standardised incidence of appendiceal cancers in all agegroups. B‐K Age‐period‐cohort analysis of colorectal cancer excluding appendiceal cancers. B Statistically significant birth cohort effects were observed with cohorts born after 1986 at 2·2 fold (95% CI 1·3‐3·8; X2 = 70·8, p < 0·001) increased risk of CRC compared to earlier cohorts. C Local drift of incidence rates are observed to be significantly different from net drift using Wald Test for heterogeneity (X2 = 36·3, p < 0·001) suggesting a single summary age‐standardised rate of CRC is inadequate to describe temporal trends in all age‐groups. D‐K Additional descriptive metrics from the age‐period‐cohort tool (National Cancer Institute's Age Period Cohort web tool, https://analysistools.nci.nih.gov/apc). Fig. S2 Age‐standardised incidence of colorectal cancer stratified by anatomical subsite: 1979 to 2015 The age‐standardised incidence of all anatomical subsites of colorectal cancer from 1979‐2015. Anatomical subsites were mapped between different ICD versions (ICD9 mapped to ICD10) for all colon and rectal codes (C18, C19 and C20). Data available from 1979 onwards as ICD8 data (1974‐1978) is not subdivided by specific anatomical site. Age‐standardised incidence is given as rate per 100 000 population. Fig S3. Age‐standardised incidence of colorectal cancer in adults aged 20‐49 years stratified by Index of Multiple Deprivation (IMD) quintile: 2001‐2015. Annual percentage change (APC) of age standardised incidence for each IMD quintile (A ‐ Least deprived to E ‐ Most deprived) and for anatomical location (proximal or distal), was calculated with an asterisk showing statisti [file BJS-107-595-s001.docx]

**BJS11486**

**Demographic trends in the incidence of young-onset colorectal cancer: a population-based study**

A. C. Chambers, S. W. Dixon, P. White, A. C. Williams, M. G. Thomas and D. E. Messenger

**Fig. S1** **Colorectal cancer and appendiceal cancer incidence trends in England: 1974 to 2015**


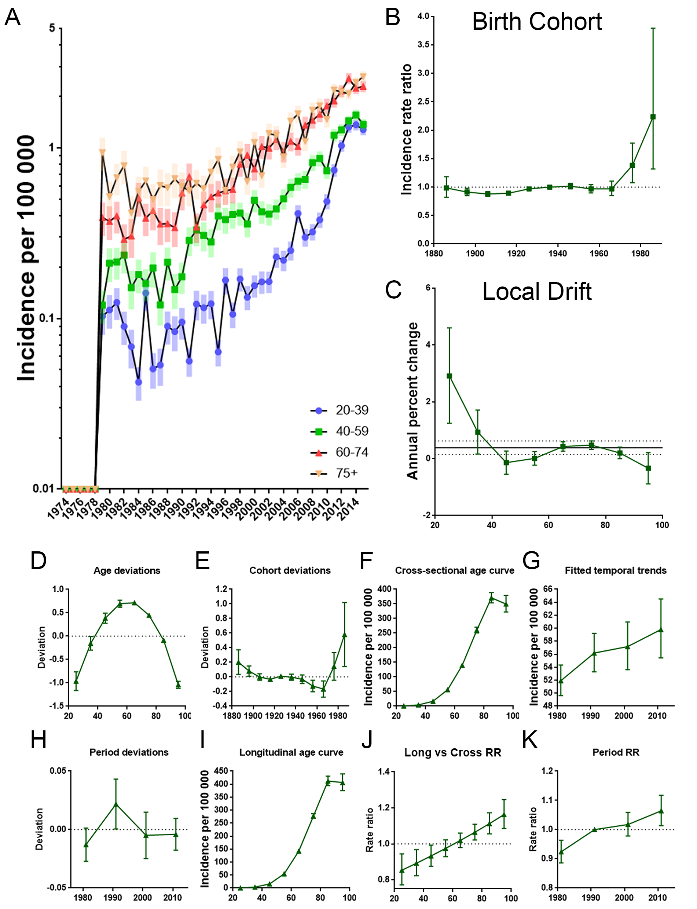


A Age-standardised rates of appendiceal adenocarcinoma in England 1979-2015. Appendix adenocarcinomas were identified using ICD9 code C153.5 (1979-1994) and the ICD10 code C181 (1995-2015) with no data available prior to 1978. Data shows increasing age-standardised incidence of appendiceal cancers in all agegroups. B-K Age-period-cohort analysis of colorectal cancer excluding appendiceal cancers. B Statistically significant birth cohort effects were observed with cohorts born after 1986 at 2.2 fold (95% CI 1.3-3.8; X2=70.8, p<0.001) increased risk of CRC compared to earlier cohorts. C Local drift of incidence rates are observed to be significantly different from net drift using Wald Test for heterogeneity (X2=36.3, p<0.001) suggesting a single summary age-standardised rate of CRC is inadequate to describe temporal trends in all age-groups. D-K Additional descriptive metrics from the age-period-cohort tool (National Cancer Institute’s Age Period Cohort web tool, https://analysistools.nci.nih.gov/apc).

**Fig. S2 Age-standardised incidence of colorectal cancer stratified by anatomical subsite: 1979 to 2015**


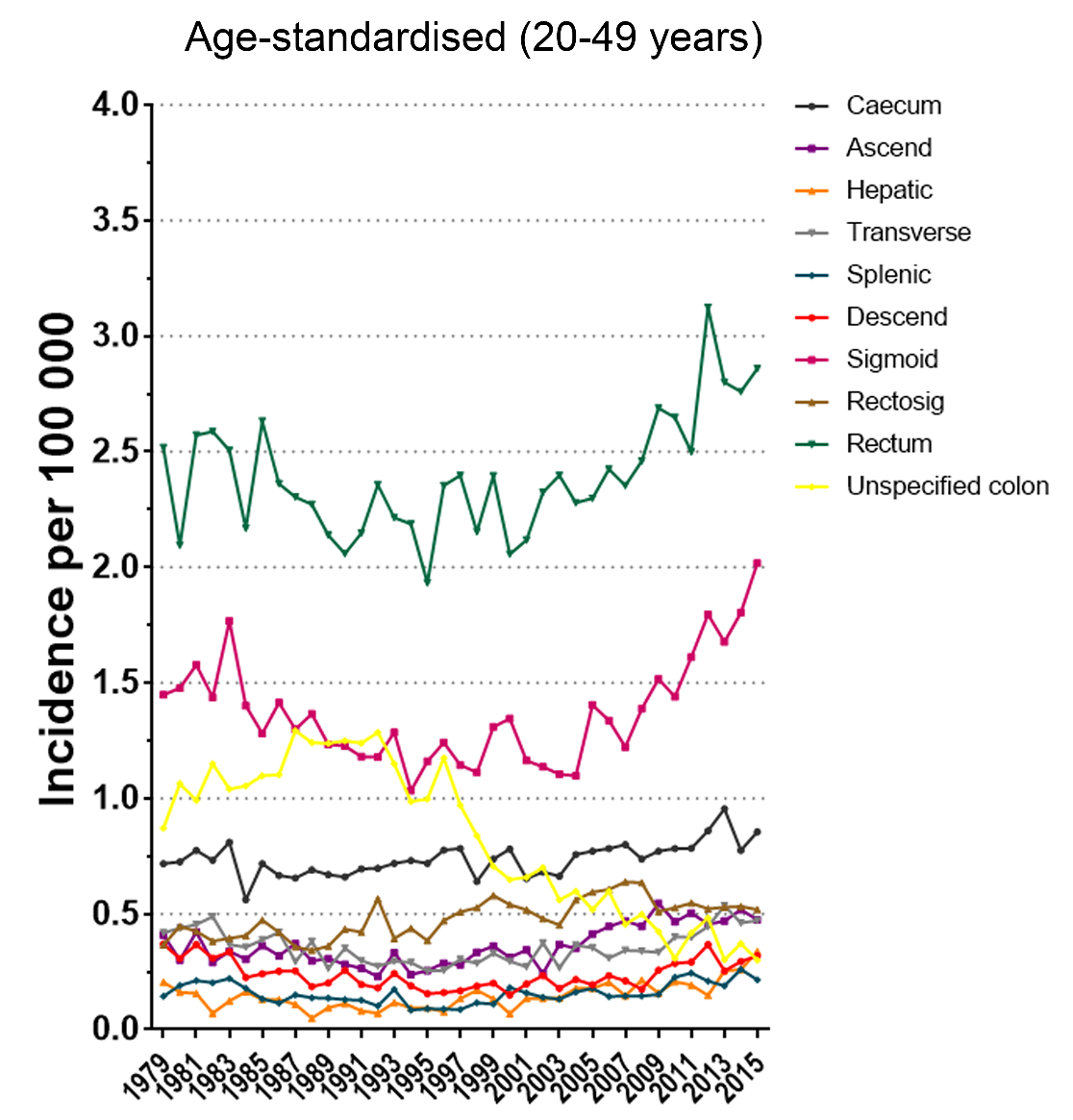


The age-standardised incidence of all anatomical subsites of colorectal cancer from 1979-2015. Anatomical subsites were mapped between different ICD versions (ICD9 mapped to ICD10) for all colon and rectal codes (C18, C19 and C20). Data available from 1979 onwards as ICD8 data (1974-1978) is not subdivided by specific anatomical site. Age-standardised incidence is given as rate per 100 000 population.

**Fig S3. Age-standardised incidence of colorectal cancer in adults aged 20-49 years stratified**

**by Index of Multiple Deprivation (IMD) quintile: 2001-2015.**


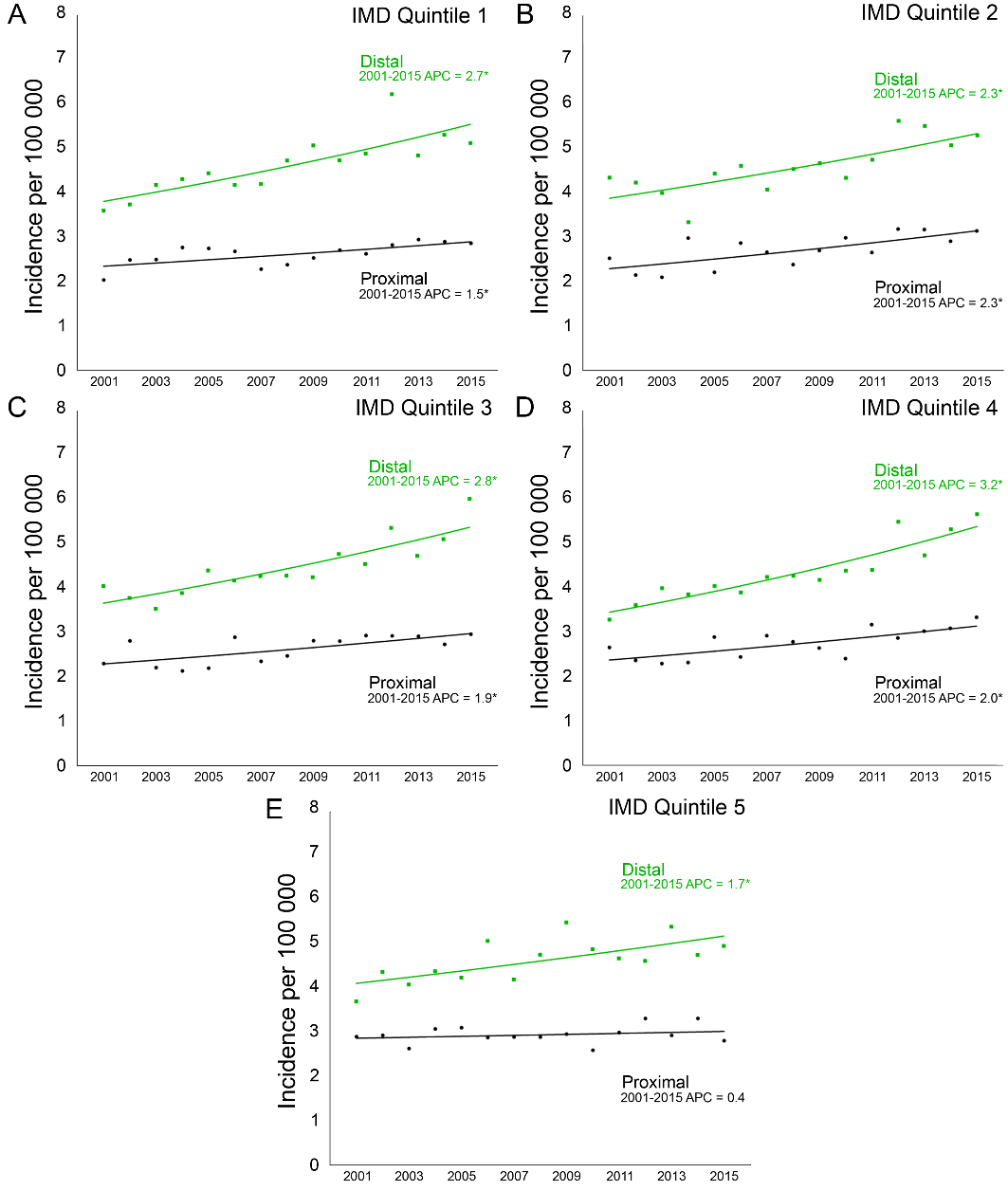


Annual percentage change (APC) of age standardised incidence for each IMD quintile (A - Least deprived to E - Most deprived) and for anatomical location (proximal or distal), was calculated with an asterisk showing statistically significant differences in APC from zero with p<0.05 using the permutation model of logarithmically transformed data. Regression lines were analysed using Analysis of co-variance (ANCOVA) and revealed no evidence of a difference in incidence rate increases between IMD quintiles for either proximal (p=0.110) or distal (p=0.230) cancers.
